# Unintended consequences of changes to Open Access and the Impact Factor

**DOI:** 10.1038/s44319-024-00172-z

**Published:** 2024-06-11

**Authors:** Frank Gannon

**Affiliations:** QIMR Berghofer Medical Research Institute, Brisbane, Australia

**Keywords:** Science Policy & Publishing

## Abstract

The move to Open Access publishing and the diminishing weight of the Impact Factor has unintended and even negative consequences.

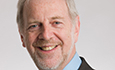

In an ideal world, scientific publications and research data should be freely available to everyone. After all, taxpayers fund most of the research, scientists use their skills to generate results and their colleagues review, refine and certify the content *pro bono*. Sharing new developments and data with all who are interested, should be the logical end point of their efforts, but a system had evolved that militated against that. Before the Open Access (OA) campaign started in earnest about 20 years ago, the fruits of the endeavors of the scientific community were available only to those located in institutions where libraries paid subscriptions to publishers, who, in turn, passed on significant profits to their shareholders. Although there were some caveats, OA promised to change that paradigm.

In an ideal world, the Impact Factor (IF) of a journal should not be a surrogate for the quality of every paper published there. The flaws of the IF have been known for some time but were largely ignored. Grant reviewers and hiring committees used the IF hierarchy of journals as a lazy shorthand for considering applications and promotions. As a result, any paper published in some high-IF journal inevitably created a more positive impression than papers from other journal titles, irrespective of the individual paper’s quality. A proliferation of subtitles from the leading brands added to the confusion. The 2012 San Francisco Declaration on Research Assessment (DORA) encouraged abandoning the use of IFs with the resultant need for committees to actually read papers and judge them by their content and not by the journal in which they were published. These two good ideas—greater OA and diminished use of IF—have made constant progress over the years. World-leading research organisations and funding agencies have pushed hard for OA while they banned the use of IF in applications for funding or promotion.

But we are not living in an ideal world. Resentment at the profits made by professional publishers was a core component of the OA movement. However, there are inevitable costs in moving a submitted manuscript from the inbox of an editor to the website of the journal. Some of these are technical and unavoidable. Others are more discretionary costs related to maintaining a team of professional editors, the website, the archive and the overall management of the journal. Of these, a team of editors is the most expensive one and the vast majority of journals work with only a managing editor and out-source the work to contractors or unpaid scientists. These costs and considerations apply both to learned societies—“not-for-profit organisations” must be also “not for loss”—as well as commercial publishers. Both are part of the real world and have to generate income either to fund their other scientific activities, such as conferences or grants, or to secure investment by shareholders. It is not surprising that professional publishers do not want to shoulder the costs of making “their” content freely available, without any financial rewards.

As the revenue has to come from somewhere, OA typically shifted the bill for a paper from the institutional subscriber to the author. While free access to the scientific literature improved, the transition to OA has not changed the overall economics of scientific publication. Perversely, OA initially just transferred the costs of publications from the institutions’ library to the much more constrained laboratory budget, which means a few thousand dollars less for reagents that are needed for the next experiments as the recurrent material budget had already been spent on the work to get the data for the paper. In other words, OA was not correcting the anomalous situation described above: the costs of scientific publication are still fully paid for by the research community—and eventually the taxpayer—and the profits are accrued by publishers. Slowly, national systems and alliances of institutions are reacting to diminish the burden on individual laboratories by negotiating with publishers more beneficial collective deals for their researchers. Surprise: this creates a new ‘subscription’ model for journals which still excludes many scientists, including clinicians and institutions or countries that chose not or cannot afford to join. We are almost back to where we started.

While the movement to OA has allowed progress to be made on breaching paywalls, it has triggered some unintended consequences. The new and evolving financial models threaten many journals from learned societies (Johnson and Malcolmson, 2024). That is a negative outcome as disciplines need to support their specialist areas and the best peer reviewing often comes from scientists committed to a society of like-minded researchers. However, a greater threat to the scientific endeavor has come from pop-up publishers who identified a new business opportunity from the combination of increasing OA and the decreasing relevance of IFs. Without IF as a guide, the name of the journal becomes largely irrelevant. In the absence of the Journal IF, an author-paid paper in a journal with low-quality standards is as visible and has the same legitimacy as one in a journal driven by high standards for its content. Moreover, these opportunistic publishers increase their profits by higher acceptance rates of submitted papers and hence a lower quality bar for publication. Weekly invitations to submit papers or join the editorial board of journals with random and often irrelevant titles are the signs of a whole segment of the industry that will accept any submission, with very minimal peer review or no review at all, provided that the author pays for it. A similar industry has grown in the area of scientific meetings where CVs can be readily bolstered by accepting invitations to be a keynote speaker at a self-proclaimed world-leading conference.

The risk of this development is that the scientific literature becomes polluted by papers that would not survive analysis in a journal club. It then becomes difficult for those outside the system, who look to “the science” for guidance on policies or treatment, to know where truth is. Contradictions and corrections are central to the scientific process and to increase knowledge. However, if papers can be published without the stamp of peer review, this scientific debate culture becomes a convenient cloak for those who use science to disrupt. The steps towards science becoming opinion-based rather than factual and shifting from objective data to a social construct are facilitated by the unhindered publication of shoddy work merely because the invoice has been paid.

The genie is out of the bottle. Reversing OA is no longer a feasible or desirable solution. The next step has to be marginalizing bad science and shady journals. In the absence of journal-based IFs, a system that focuses on the articles is needed. The Relative Citation Ratio (RCR) (Hutchins et al, 2016) provides a mechanism for time- and discipline-dependent citations to be calculated and could—and perhaps should—be tagged to papers that are part of grant or promotion assessments. Just as packaging of food carries information for the consumer, journals should also indicate the standard of the peer review they conduct and their quality checks. Information on their systems for detecting image or data manipulation, plagiarism, AI-generated papers, or papermill fictions should become a requirement to indicate their quality standards. Broad bands based on these criteria could be used to categorize journals. Applicants who inflate their CVs with ‘bought’ publications should not be seen as equivalent to those who subject their work to the highest standards of rigor.

There is no ideal world. The Utopia of full OA and no IF has been polluted. As a result, we need to do better quickly to protect those parts of the publication system that are good and beneficial to society if we wish to maintain the credibility of science.

### Supplementary information

Peer Review File
