# Supplementary material for: Unintended consequences of changes to Open Access and the Impact Factor
Source: EMBO Rep. 2024 Jun 11;25(7):2815–6. doi: 10.1038/s44319-024-00172-z (PMC11239826; doi:10.1038/s44319-024-00172-z)
Supplement: Supplementary file 1 — Peer Review File [file 44319_2024_172_MOESM1_ESM.pdf]

EMBO reports

Unintended consequences of changes to Open Access and the Impact Factor

Frank Gannon

Corresponding author(s): Frank Gannon (FGannon@qimrberghofer.edu.au)

Review Timeline:

Submission Date:	31st May 24
Accepted:	3rd Jun 24

Editor: Holger Breithaupt

Transaction Report: This article type is not peer reviewed.
